# Feshbach Resonances in Cold Collisions: Benchmarking State-of-the-Art Ab Initio Potential Energy Surfaces

**DOI:** 10.1021/acs.jpclett.5c01581

**Published:** 2025-07-28

**Authors:** Karl P. Horn, Meenu Upadhyay, Baruch Margulis, Daniel M. Reich, Edvardas Narevicius, Markus Meuwly, Christiane P. Koch

**Affiliations:** † Freie Universität Berlin, Dahlem Center for Complex Quantum Systems and Fachbereich Physik, Arnimallee 14, 14195 Berlin Germany; ‡ Department of Chemistry, 27209 University of Basel, CH-4056 Basel, Switzerland; ¶ Department of Chemical and Biological Physics, Weizmann Institute of Science, 7610001 Rehovot, Israel; ∥ Department of Physics, Technische Universität Dortmund, 44227 Dortmund, Germany

## Abstract

High-quality potential energy surfaces (PESs) are prerequisites for quantitative atomistic simulations with both quantum and classical dynamics approaches. The ultimate test for the validity of a PES entails comparisons with judiciously chosen experimental observables. Here we ask whether cold collision measurements are sufficiently informative to validate and distinguish between high-level, state-of-the art PESs for the strongly interacting Ne–H_2_^+^ system. We show that measurement of the final state distributions for a process that involves several metastable intermediate states is sufficient to identify the PES that captures the long-range interactions properly. Furthermore, we show that a modest increase in the experimental energy resolution will allow for resolving individual Feshbach resonances and enable a quantitative probe of the interactions in the short and intermediate ranges.

## Graphical abstract

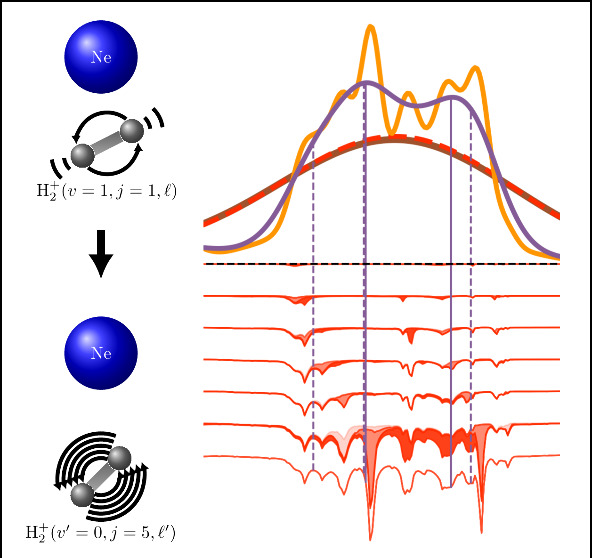

Collision experiments measure the probability that collision partners will change their internal state(s) or undergo a chemical reaction.^1^ Since collisions ultimately probe interparticle interactions, comparing measured and calculated collision cross sections allows us to quantify, in principle, how well our theoretical understanding matches physical reality in the experiments.^2,3^ For molecular collisions, however, such a quantitative assessment of theoretical models has long been hampered by the stringent requirement that experiments need to resolve both initial and final states.^4,5^ Investigating cold and ultracold collisions provides a means to prepare well-defined initial states with quantum purity.^6^ Final-state resolution has recently been added to these experiments by collecting the reaction products with velocity map imaging.^7–10^ But even with state-to-state resolution, it is often challenging to identify suitable experimental observables that provide information on local or global properties of the underlying potential energy surface (PES).^11^ This is true in particular for collision systems, where the interaction is strong enough to couple different internal and external degrees of freedom.^7,12–14^

For molecular collisions, the interaction between the partners is fully described by the PES. How much information about a PES can be inferred from measurements depends on characteristics such as the range and degree of anisotropy of the intermolecular interactions. For example, an almost fully statistical distribution over the energetically allowed final states was observed in reactive collisions with very strong anisotropy,^7^ even though the collisions proceed in the fully quantum regime. In contrast, clear quantum fingerprints in the cross sections have been observed in reactive collisions between rare gas atoms and dihydrogen molecules, even though the interaction is similarly anisotropic.^8^ This difference points to the importance of resolving not only initial and final but also intermediate quantum states of a collision.

Quantum scattering calculations for atom–diatom complexes, as considered here, can be fully converged. Thus, the computed observables depend only on the quality of the underlying PES, which is determined by the electronic structure method and numerical technique used to calculate and represent it. Changes in the shape of the PES will directly translate into modifications of the observables, which can be exploited, for example, by “PES morphing” through suitable coordinate transformations.^15–17^ At the same time, a given physical observable is usually sensitive only to specific regions and properties of a PES. For example, Feshbach resonances (FRs) are particularly sensitive to and informative about the long-range part of a PES but do not directly provide information about the well depth. Even with an exhaustive set of measured FRs, not all properties of a full-dimensional PES can be probed and adjusted.^16^

In the present contribution, we invert this perspective by asking: Can we distinguish the quality of different PESs and learn about their merits and deficiencies? By how much does the experimental resolution need to be improved to discriminate between different PESs? The proxy for addressing these questions is the Ne–H_2_^+^ complex, for which an exhaustive list of measured FRs is available.^8^ These metastable intermediate states probe wide parts of the PES;^16^ they are ubiquitous in few-body reaction dynamics^18–22^ and were shown to lead to unique quantum fingerprints in the final state distribution.^8^ For Ne interacting with H_2_^+^(1^2^A′), full-dimensional electronic structure calculations in the current “gold standard” CCSD­(T) (coupled cluster with single, double and perturbative triples) method^23^ using quintuple-zeta quality basis sets have been carried out.^8^ In contrast, full CI calculations are computationally too costly, but earlier PESs at the multireference configuration interaction (MRCI) are available.^24,25^ In the following we will compare three PESs which differ, in addition to the electronic structure method, in terms of utilized basis sets (quadruple vs quintuple) as well as sampled grid points and interpolation methods.

Experimentally, the dynamics on the ionic PES are initiated by Penning ionization^26^ following collision of a metastable Ne* with H_2_. The collision on the neutral surface, prior to the Penning ionization, can be regarded as the “first half” of a process proceeding in two distinct steps.^27,28^ On the neutral surface, the initial state is a plane wave characterized by its energy. The second “half-collision”, on the ionic surface, starts with an initial wavepacket, with an approximately Gaussian shape, and a mean Ne–H_2_^+^ separation of ∼9a_0_. In between the two half-collisions, Penning ionization is modeled as a vertical transition to the electronic ground state of the molecular ion,^8,29^ populating different vibrational states of Ne–H_2_^+^(*v,j*). These decay to Ne + H_2_^+^(*v′,j*′) and yield the H_2_^+^(*v′,j*′) translational kinetic energy spectrum as the main observable. The pronounced angular anisotropy of the Ne–H_2_^+^ interaction leads to rovibrational quenching for *v* > 0, which converts vibrational into rotational and kinetic energy. This is reflected in distinct peaks in the H_2_^+^ kinetic energy spectrum,^8^ which correspond to different final *j*-rotational states. Each measured peak consists of contributions from different total angular momenta J∈{|l−j|,...,l+j} and partial waves l^8^ and, most importantly, several FRs which, unlike the final rovibrational states, cannot be easily resolved in the experiment but are amenable to computations. Such “quantum fingerprints” of the collision dynamics are partially averaged out in the experiments due to the finite energy resolution of the detector.

To assess the quality of the three PESs considered (CCSD­(T)-5, MRCI-5, MRCI-4; see Supporting Information), full coupled-channel quantum scattering calculations were carried out. The physical system can be described using the three-dimensional Jacobi coordinates *R*, *r*, and θ, where *R* describes the distance between Ne and the H_2_^+^ center of mass, *r* is the H_2_^+^ bond length, and θ is the angle between the H_2_^+^ axis and the axis connecting Ne with the H_2_^+^ center of mass. The total energy of the system, given as the sum of kinetic and internal energy, is conserved; its zero is chosen to correspond to the dissociation limit of the input channel, i.e., the channel of the initial wave packet, which depends on *v* and *j*. The kinetic energy is given in terms of the relative momentum of the two particles’ center of mass frame, ${Ê}_{kin}=\nabla_{R}^{2}/2\mu$ . The topography of the PESs considered^8,24,25^ is shown in the Supporting Information, with MRCI-4 significantly deviating from CCSD­(T)-5 and MRCI-5 in terms of minimal well depth but also angular anisotropy. In terms of long-range behavior, CCSD­(T)-5 agrees well with the behavior expected for the electrostatic interaction between a singly charged ion and a neutral particle,^31^ whereas MRCI is generally known to have difficulty in properly predicting the long-range behavior, which is also seen here for MRCI-5 and MRCI-4. In contrast, at short range, differences between CCSD­(T)-5 and MRCI-5 are rather small. Calculation of the cross sections uses the computational framework detailed already in earlier work.^8^ Briefly, the total angular momentum *J⃗* of the collision complex is conserved, as is parity. Here J⃗=L⃗+j⃗ , where *j⃗* describes molecular rotation and *L⃗* the rotation around the center of mass of the collision complex. Rovibrational quenching leads to changes in the partial wave quantum number l , in order to preserve *J* (with J∈{|l−j|,...,l+j} ). The basis for the coupled channels calculations is thus characterized by quantum numbers *J*, *M_J_*, *j*, l , and *v*,^8^ and each of the potentials is expanded in this basis.

Figure 1 shows the translational kinetic energy spectra obtained with the three PESs for initial wavepackets with *v* = 1 and convoluted with the experimental resolution. The spectra are shifted along the kinetic energy axis to minimize the root-mean-square (RMS) difference between the computed and observed peak positions. Analogous data for *v* = 2 is shown in the Supporting Information. Without shifting, the peak positions can differ by up to 30 cm^–1^, see Table 1. At this stage, the quality of the CCSD­(T) PES is superior to that of the two MRCI-based PESs. However, it should also be noted that MRCI-5 and MRCI-4 are reactive PESs and were developed to investigate proton transfer between H_2_^+^ and Ne. The peak positions quantify the amount of internal energy that was converted into kinetic energy and, thus, directly reflect the energy of the FRs. Once this source of deviation between prediction and measurement is accounted for, the remaining comparison focuses on the distribution of the converted energy over the different final rovibrational states, see Figure 1(a)-(c). The latter is mainly determined by the angular anisotropy. Given the experimental resolution, all three PESs compare reasonably well with the experimental data. The broad peaks in Figure 1(a)-(c), most of them well separated in *v*′ and *j*′, correspond to the different final state contributions; i.e., it is almost always possible to resolve the final H_2_^+^(*v′,j*′) states with the current experimental resolution.^8^ In contrast, while without convolution most of the peaks in the kinetic energy spectra can be attributed to a specific initial Feshbach resonance state (with *J*, l , and *j*, shown in light shade), this information is lost after convolution. The bare, i.e., unconvoluted, cross sections display much larger differences between the PESs.

**Figure 1:**
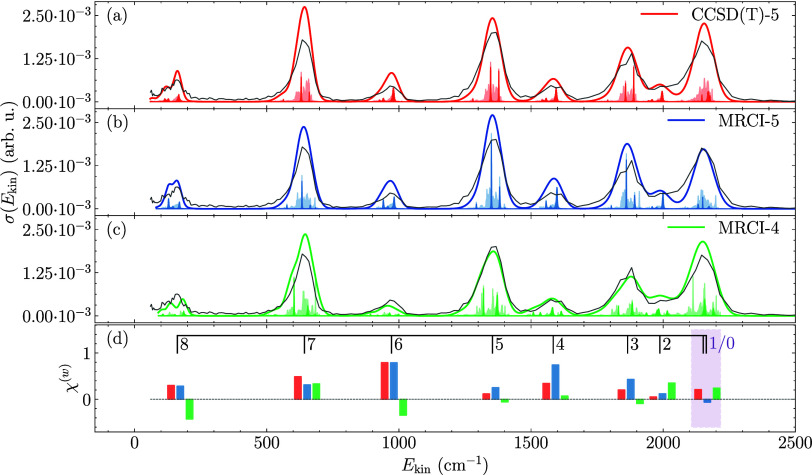
(a)-(c) Calculated cross sections (summed over all initial total angular momenta *J*, partial waves l , and product *v*′, *j*′, l ′ and convoluted with the detector resolution) as a function of the kinetic energy, compared with the experimental data^8^ (gray) for an initial wavepacket in the diatomic rotational ground state and with *v* = 1 contrasted with its unconvoluted form (scaled to one-tenth of its relative height, lightly shaded areas). Darkly shaded areas indicate the dominant total angular momentum and partial wave combinations l = 5 and *J* = 5 for both *para*- and *ortho*-H_2_^+^. (d) Integrated deviations χ^(w)^, cf. eq (S4), using the same color coding. The window highlighted in purple comprises two final *j*′ contributions, as indicated by the comb.

**Table 1:**
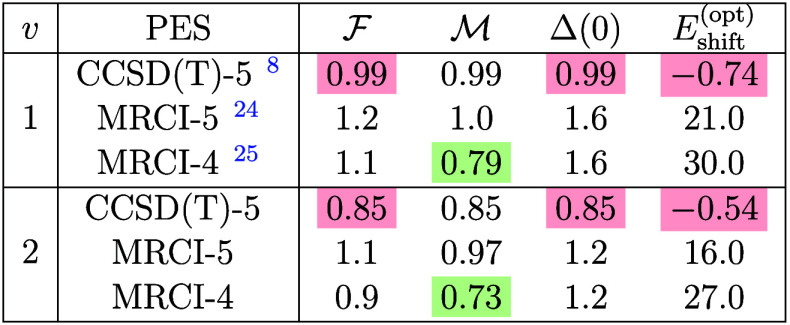
Agreement between Calculated and Experimental Cross Sections in Terms of F , cf. Eq (S3), the RMS Deviation Obtained for the Energy-Shifted Spectrum M , cf. Eq (S2), the RMS Deviation for the Unshifted Spectrum Δ(0), and the Optimal Energy Shift *E*_shift_^(opt)^ ^a^

a Values are reported with normalization according to eq (S1) and in multiples of 10^–2^ except for *E*_shift_^(opt)^ in cm^–1^. The PES performing best for a given quantifier is highlighted with the color code of Figure 1.

To quantify the agreement for the peak positions, we introduce the energy *E*_shift_^(opt)^, by which the spectra have to be shifted to optimally match the experimental peak positions. This shift has to be compared to the (velocity-dependent) experimental uncertainty in the peak positions, which is ∼20 cm^–1^ for the low-*j*′ peaks but only 2 cm^–1^ for the high-*j*′ peaks. The smallest shift by far is needed for the cross sections obtained with the CCSD­(T)-5 PES, see Table 1. To quantify the matching in terms of the peak heights, the (signed) differences χ^(w)^ are evaluated for an energy window “w” to assess the agreement on a per-peak basis, see Figure 1(d). This confirms the comparable performance of all three PESs in terms of the peak heights, in particular for the l strong peaks. Finally, a single figure of merit F was obtained by averaging the RMS difference with respect to the energy shift up to the optimal value *E*_shift_^(opt)^, see the Supporting Information. This figure of merit is designed to reward PESs that correctly predict the energies at which the Feshbach resonances occur while also ensuring that the distribution of peak intensities is correct. In terms of F , the CCSD­(T)-5 PES yields the closest match with the experimental data for both *v* = 1 and 2, cf. Table 1. This is mainly due to the small energy shift *E*_shift_^(opt)^ ≤ 1 cm^–1^ required.

The advantage of the CCSD­(T)-5 PES derives from the fact that the RKHS representation of the CCSD­(T) energies results in more accurate positions of the FRs. Focusing on the cross section as a function of the total energy allows for a more in-depth analysis of the FRs, cf. Figure 2. As a function of total energy, both peak shapes and positions of the cross sections differ vastly among the PESs. In particular, a clear bias toward lower energies is seen for MRCI-5 and MRCI-4 as compared to CCSD­(T)-5. This is true for both *v* = 1 and *v* = 2 (shown in the Supporting Information) and *para*- as well as *ortho*-H_2_^+^. For *para*-H_2_^+^ the cross sections for both *v* = 1 and *v* = 2 are primarily due to l = 5, *J* = 5 (shown with dark shade) and are comprised of three main peaks, corresponding to well-isolated FRs. In contrast, for *ortho*-H_2_^+^, where the dominant partial wave contribution consists of three different total angular momenta *J*, the cross sections indicate several, partially overlapping FRs, cf. Figure 2 (e)-(g) for *v* = 1 (the data for *v* = 2 is shown in Figure S5­(d)-(f)). Focusing on *v* = 1 for *para*-H_2_^+^, Figure 2 (a)-(c), our analysis is facilitated by the fact that a single resonance, around *E* = −17 cm^–1^, dominates for CCSD­(T)-5, while the cross sections obtained with MRCI-5 and MRCI-4 are both comprised of two significant contributions, occurring at lower energies, around −30 cm^–1^ and −70 cm^–1^, respectively.

**Figure 2:**
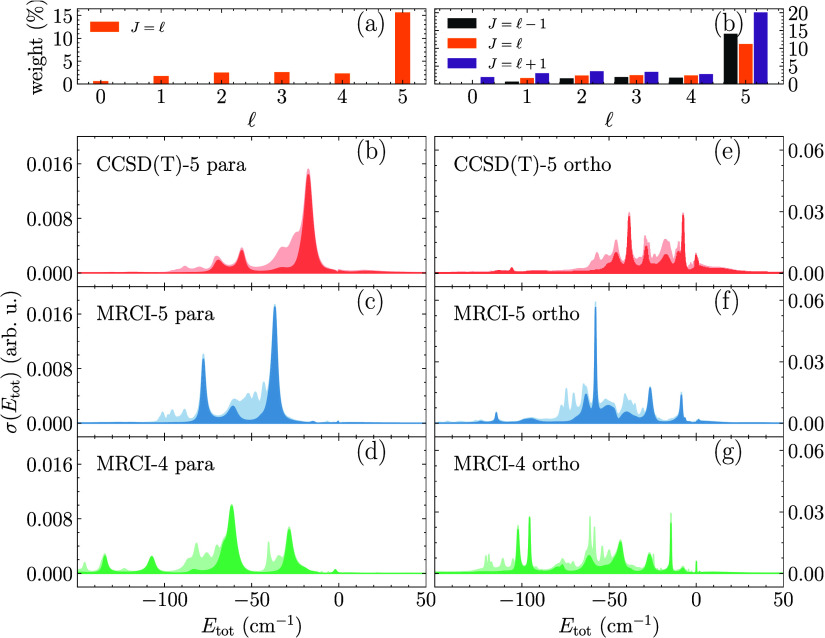
Calculated collision cross sections (b-d, e-g) as a function of the total energy. The top set of panels (a,b) shows the weighting of initial total angular momentum *J* and partial wave l contributions. In the bottom set of panels, (b-d) correspond to *v* = 1 and *para*-H_2_^+^ with initial *j* = 0, while (e-g) correspond to *ortho*-H_2_^+^ with initial *j* = 1. The lightly shaded backgrounds denote the total spectra, while the specific contributions due to the (dominant) initial l = 5 partial wave are portrayed as darkly shaded slabs.

The cross section peaks in Figure 2 are linked to the topology of the PES and the shapes of the resonance wave functions. At small *R* the MRCI-4 PES differs from the CCSD­(T)-5 and MRCI-5 PESs, especially by its much deeper well (see the Supporting Information). The squared amplitudes of the most important resonance wave functions are shown in Figure 3, where the left set of panels also compares them with the “initial” wave packet (gray shaded curve), i.e., *v* = *v*′ = 1, *j* = *j*′ = 0, and l = l ′ = 5. The wave function amplitude covers a large range of interparticle distances, from the strongly interacting region (*R* ≈ 3.4a_0_ to 3.8a_0_, depending on the PES) where the anisotropy is most pronounced, all the way to long-range interactions. Here it becomes apparent that the (main) resonances for CCSD­(T)-5 and MRCI-5 show significant overlap with the input wavepacket. In contrast, for MRCI-4, the two main resonances are centered around slightly shorter or larger, respectively, distances as compared to the input wavepacket. They also have much larger amplitudes relative to those of the other two surfaces. The right set of panels in Figure 3 demonstrates the angular behavior of these resonance wave function components by taking the partial wave quantum number into account. The similar topologies of the CCSD­(T)-5 and MRCI-5 PESs at short range correspond to their peak intensities matching each other more closely than those obtained with the MRCI-4 PES, even though the energies at which these resonances occur are appreciably different, cf. Table 1. In contrast, for large *R* the CCSD­(T)-5 PES differs most, while the two MRCI PESs show similar long-range behavior. The best performance of CCSD­(T)-5 in terms of *E*_shift_^(opt)^, combined with the fact that the two MRCI PESs demonstrate similar long-range behavior as well as comparable values for *E*_shift_^(opt)^, suggests that the long-range behavior of the PES primarily determines the peak positions. This confirms earlier findings from quantum wavepacket simulations for half-collisions between He and H_2_^+^ molecules.^16^

**Figure 3:**
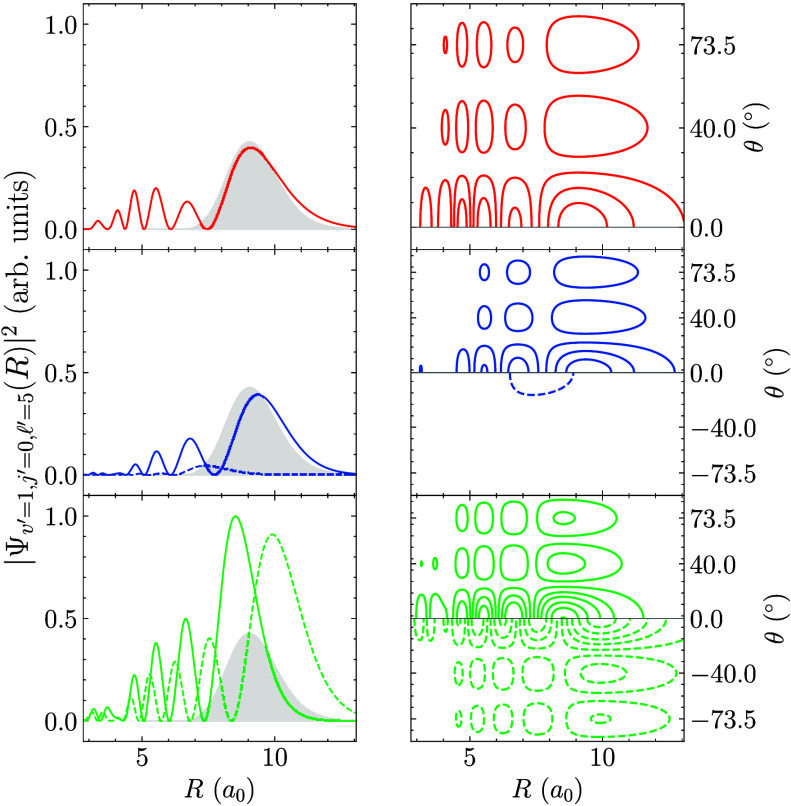
Components of the resonance wave functions’ squared amplitude with *v*′ = 1, *j*′ = 0, and l ′ = 5, cf. Figure 2(a-c). The resonance energies are −17.45 cm^–1^ for CCSD­(T)-5 in red, −36.75 cm^–1^ (solid) and −77.65 cm^–1^ (dotted) for MRCI-5 in blue, and −61.45 cm^–1^ (solid) and −28.45 cm^–1^ (dotted) for MRCI-4 in green. Where applicable, the two highest resonances are shown in the positive and negative angular domains of the contour plots, respectively. The shaded gray areas show the shape of the initial wave function on the ionic surface.

The conspicuously substantial long-range anisotropy of the MRCI-4 PES and the resulting difference in wave functions and cross sections indicate that the peak heights in the final *v*′,*j*′-distribution are particularly sensitive to the potential at short and intermediate *R*. This is where many avoided crossings between the adiabatic potential energy curves for each *v*,*j* (resulting from an adiabatic separation of vibrational and rotational motion) are observed.^8^ Passage through the crossings redistributes the energy to the various final *v*′ and *j*′ states, which in turn is reflected in the peak height of the cross sections.

The figure of merit F for the CCSD­(T)-5 PES is lower by 5% to 20%, respectively, compared with those of the MRCI-5 and MRCI-4 PESs, depending on the vibrational state *v* considered, see Table 1. Based on this, we now address the question by how much the resolution of the experiments needs to be improved in order to resolve individual l components to further validate the PESs. To this end, kinetic energy spectra for three different experimental resolutions—the current value as well as 4-fold and 10-fold improved resolution—are compared in Figure 4. For better visibility, we focus on one exemplary peak: *j*′ = 8 for *ortho*-H_2_ in the right and *j*′ = 5 for *para*-H_2_ in the left part of Figure 4. When scaling the resolution, the convolution width is assumed to be independent of the kinetic energy, whereas a kinetic energy-dependent convolution width accounts for experimental uncertainty.^8^ Figure 4 shows the convoluted final state distribution at the present experimental resolution^8^ with fixed convolution widths (as opposed to being energy-dependent) for better comparability, along with the curves for the 4-fold and 10-fold improved resolutions (with fixed widths). The differences between the fixed-width and energy-dependent convolution widths are minor.

**Figure 4:**
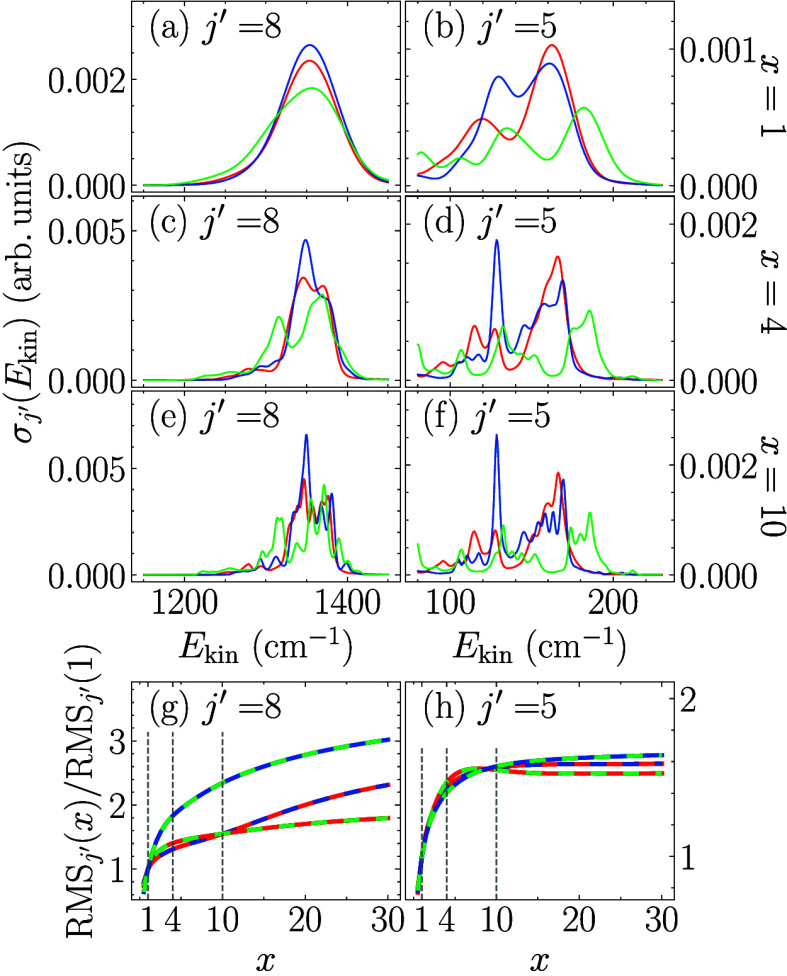
Comparison of the cross sections calculated with three different PESs for increasing experimental resolution *x* and *j*′ = 8 (a,c,e) and *j*′ = 5 (b,d,f) and change in RMS deviation for each pair of PESs (indicated by the color coding) as the resolution is enhanced (g,h). Same color for PESs as before.

At 4 times the current experimental resolution, the l substates can be identified and the convoluted spectra display a different energy dependence for the different PESs. For the contribution of the dominant initial partial wave ( l = 5) and in some cases also for the initial partial waves with lower weight, individual peaks in the spectra can be attributed to specific FRs occurring at different energies, for example the peaks at kinetic energies of 175 cm^–1^ and 625 cm^–1^. In the case of CCSD­(T)-5, three distinct peaks are seen at roughly 115 cm^–1^, 130 cm^–1^, and 170 cm^–1^ in Figure 4(d); they correspond to the three peaks at −70 cm^–1^, −55 cm^–1^, and −20 cm^–1^ in Figure 2(a). For MRCI-5, two pronounced peaks are seen at 130 cm^–1^ and 170 cm^–1^ along with a flatter peak at roughly 140 cm^–1^ in Figure 4(d), which can be attributed to the various peaks occurring at about −80 cm^–1^, −60 cm^–1^, and −35 cm^–1^ in Figure 2(b). MRCI-4, in contrast, displays broad peaks at around 110 cm^–1^, 150 cm^–1^, and 185 cm^–1^ in Figure 4, corresponding to the −110 cm^–1^, −60 cm^–1^, and −30 cm^–1^ peaks in Figure 2(c). To conclude, enhancing the energy resolution by a factor of 4 has a two-fold effect: On top of deciding which of the PESs best captures the details of the interaction, it will also allow for assigning the peaks in the kinetic energy spectrum to specific FRs.

At 10 times the current experimental resolution, the splitting is much more pronounced and several peaks can be clearly attributed to different initial *J*, l channels. Most importantly, however, the collision complex features sufficiently many, energetically well-isolated FRs such that the shapes of the convoluted cross sections differ in a pronounced fashion among the three PESs. This is discussed in more detail in the Supporting Information.

The largest gain in improving the ability to differentiate the PESs is observed when increasing the resolution by a factor of 4. In order to make this observation more quantitative, Figure 4(g,h) shows the peakwise RMS deviation between pairs of simulated spectra (indicated by the color code) integrated over the respective energy windows shown in Figure 4(a-f). A large increase in the RMS deviation corresponds to an increase in the ability to distinguish two theoretical predictions from each other, and thus also their respective comparison with the experimental data. As one would expect, increasing the resolution will not lead to an improved distinguishability indefinitely. At which resolution saturation sets in depends on the specific peak, i.e., the final *j*′ values: While no substantial further improvement is observed in Figure 4(h) when increasing the energy resolution by more than a factor of 10, in Figure 4(g) the distinguishability continues to increase gradually even until 30 times the original resolution.

While a 10-fold increase in the kinetic energy resolution compared to the recent experiment^8^ may prove very challenging, a 4-fold increase will require only moderate changes to the existing setup. With the corresponding kinetic energy resolution, it will already be possible to attribute a good part of the kinetic energy spectrum to specific initial and final states, taking the experiment a big step toward fully resolved “quantum tomography” of the collision. This will come on top of the ability of the measurement to decide which level of theory for the interparticle interactions captures physical reality best.

## Supplementary Material



## Notes

Visualization of PES, details of the figures of merit for comparison, calculated cross sections for an initial wavepacket with *v* = 2, scaling of the convolution resolution for additional peaks, and breakdown of individual partial wave contributions (PDF)

## @

National Institute of Standards and Technologies, Boulder, Colorado 80305, United States
