# Supplementary material for: Feshbach Resonances in Cold Collisions: Benchmarking State-of-the-Art Ab Initio Potential Energy Surfaces
Source: J Phys Chem Lett. 2025 Jul 28;16(31):7862–7. doi: 10.1021/acs.jpclett.5c01581 (PMC12337136; doi:10.1021/acs.jpclett.5c01581)
Supplement: Supplementary file 1 [file jz5c01581_si_001.pdf]

Supplementary Information:
Feshbach Resonances in Cold Collisions: Benchmarking State of the Art *ab initio* Potential Energy Surfaces

Karl P. Horn, Meenu Upadhyay, Baruch Margulis, Daniel M. Reich, Edvardas Narevicius, Markus Meuwly, and Christiane P. Koch*

I. POTENTIAL ENERGY SURFACES

The three PESs differ in the underlying approximations involved in the electronic structure methods (CCSD(T) vs MRCI), the basis sets used (aug-cc-pV5Z, indicated by the suffix '-5', and aug-cc-pVQZ, indicated by the suffix '-4' in the following), and their representations (reproducing kernel Hilbert space vs. parametrized fits). The CCSD(T)-5 PES is a reproducing kernel Hilbert space [1] (RKHS) representation of CCSD(T)/aug-cc-pV5Z reference energies computed using MOLPRO [2] for a grid of Jacobi coordinates (R, r, θ). Here, r is the H_2^+ diatomic bond length, R is the distance between the center of mass of the diatomic molecule and the neon atom and θ is the angle between $\vec{r}$ and $\vec{R}$. The grid (on-grid points) included 39 points for $r \in [1.1\,a_0, 8.0\,a_0]$, 49 points for $R \in [1.0\,a_0, 45.0\,a_0]$ and 10 Gauss-Legendre quadrature points for $\theta \in [0°, 90°]$.[3] The root mean squared errors (RMSE) for energies of 250 geometries that were not used in constructing the RKHS representation (off-grid points) between reference calculations and the evaluated RKHS is 11.8 cm^{-1}, compared with an RMSE of 11.3 cm^{-1} for on-grid points. [3] This compares with RMSEs of 94 cm^{-1} (0.27 kcal/mol) and 315 cm^{-1} (0.0391 eV) for the MRCI-5 and MRCI-4 PESs, respectively.[4, 5]

Figure S1 shows a two dimensional slice in θ and R of the various potential energy surfaces at $r = 2.0\ a_0$, which is near to the H_2^+ equilibrium distance. At short range there is close agreement between CCSD(T)-5 and MRCI-5 for all angles θ, whilst MRCI-4 shows large deviations (both radially and angularly) from the other two that grow larger in the vicinity of the minimum around $R \approx 3.4\ a_0$.

In contrast, Fig. S2 and Fig. S3 demonstrate the long range behaviour of the potential in terms of the Legendre expansion coefficients $V(R, r, \theta) = \sum_{\lambda=0}^{\infty} V_\lambda(R, r) P_\lambda(\cos\theta)$ at $r = 2.0\ a_0$, which is near to the equilibrium distance of H_2^+. We observe that as the interparticle distance R increases, the isotropic term $\lambda = 0$ of the CCSD(T)-5 potential energy surface shows a large deviation from both of the other potential energy surfaces.

II. FIGURES OF MERIT

To compare cross sections with each other, the theoretical and experimental cross sections shown above have been 'normalised' according to

$$a \int_{\mathbb{R}_+} dE_{\text{kin}} \tilde{\sigma}_{\text{exp}}(E_{\text{kin}}) = b \int_{\mathbb{R}_+} dE_{\text{kin}} \tilde{\sigma}_{\text{theory}}(E_{\text{kin}}) = 1 \tag{S1}$$

for each potential energy surface. In Eq. (S1), $\tilde{\sigma}_{\text{exp}}(E_{\text{kin}})$ and $\tilde{\sigma}_{\text{theory}}(E_{\text{kin}})$ are the cross sections prior to normalisation, whilst their rescaled counterparts appear without tildes. We discuss potential drawbacks of this scaling method and introduce an alternate method below. In particular, we show how this leads to a better overall agreement in terms of RMS deviation, but at the cost of an increase in the complexity.

When quantifying how well the calculated cross sections match the experimental cross section, we separate the mismatch in Feshbach resonance energies from the mismatch in the final state distribution, i.e., the amplitudes of the peaks corresponding to a given v' and j'. For each potential energy surface we find the most favourable E_{shift} by solving the minimization problem,

$$\min_{E_{\text{shift}}} \Delta(E_{\text{shift}}) \tag{S2}$$

$$= \min_{E_{\text{shift}}} \sqrt{\int_{\mathbb{R}_+} dE_{\text{kin}} \left|\sigma_{\text{exp}}(E_{\text{kin}}) - \sigma_{E_{\text{shift}}}(E_{\text{kin}})\right|^2}\ ,$$

with

$$\sigma_{E_{\text{shift}}}(E_{\text{kin}}) = \sigma_{\text{theory}}(E_{\text{kin}} + E_{\text{shift}})$$

the energy shifted cross section, using the NLopt python package [6]. $\Delta(E_{\text{shift}})$ in Eq. (S2), is the root-mean-square difference between the (energy-shifted) theoretical and experimental cross sections, which quantifies the error in the distribution of peak heights and shapes. We will refer to the optimal shift (in terms of the best match of peak positions) as $E_{\text{shift}}^{(\text{opt})}$.

Neither $\Delta(E_{\text{shift}} = 0)$, which emphasises the correctness of the Feshbach resonance energies over the correctness of peak amplitudes, nor $\Delta(E_{\text{shift}}^{(\text{opt})})$, which emphasises the correctness of the peak amplitudes over the peak positions, alone can fully characterize the agreement between calculated and experimental cross sections. For a fair comparison, we combine $E_{\text{shift}}^{(\text{opt})}$ and the root-mean-square deviation into a single figure of

* christiane.koch@fu-berlin.de

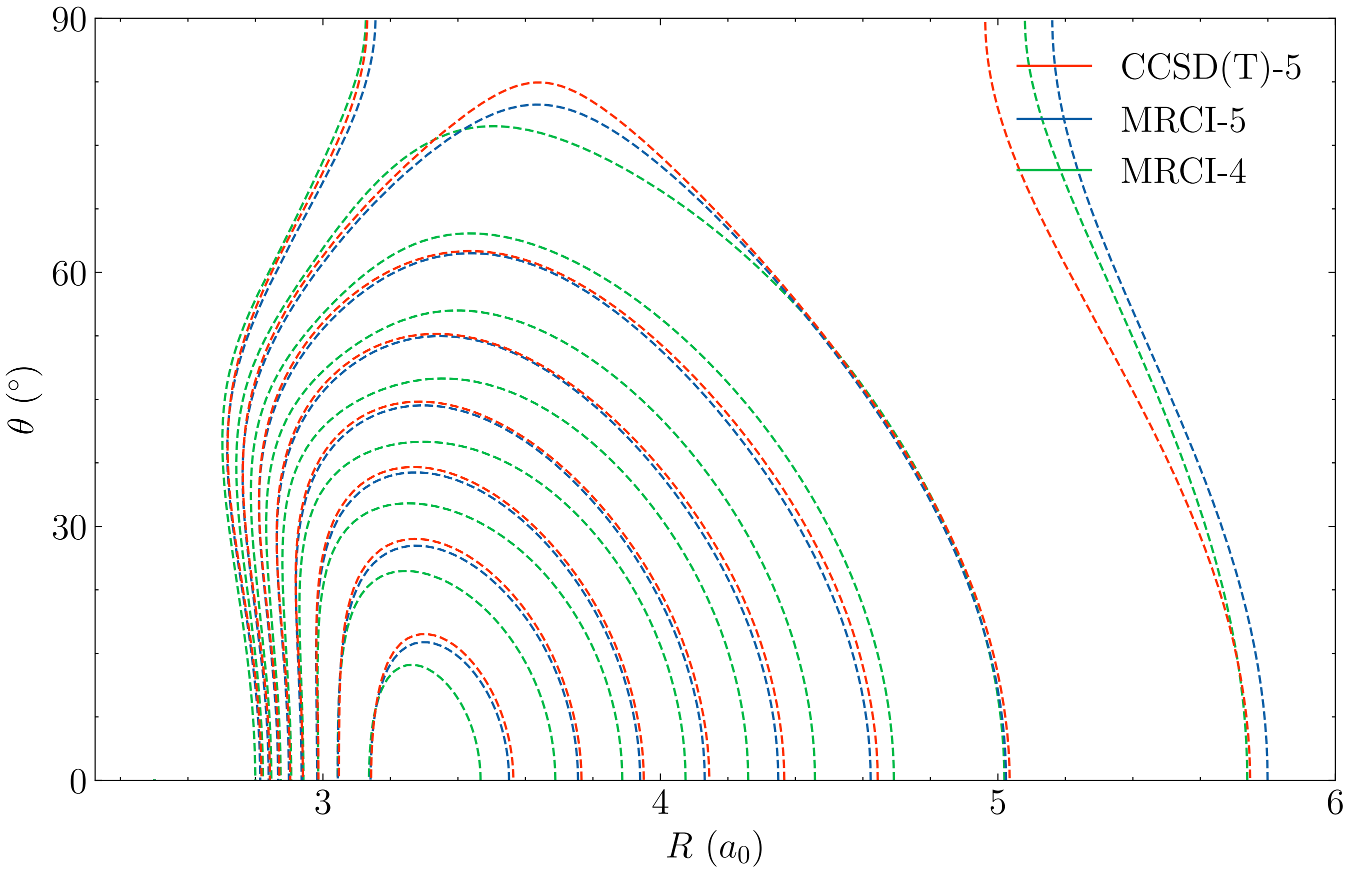

FIG. S1. Comparison of potential energy surfaces for a specific slice of coordinate space ($r = 2.0\,a_0$) in the strongly interacting, bound state region. Each potential energy surface has the same set of contours (starting at $-500\,\mathrm{cm}^{-1}$ around $R \approx 5.8\ a_0$ and decreasing in steps of $500\,\mathrm{cm}^{-1}$ towards the minimum at $R \approx 3.3\,a_0$ (note that the MRCI-4 surface has an additional contour corresponding to $-4500\,\mathrm{cm}^{-1}$, which is lower than the minimal energies of CCSD(T)-5 and MRCI-5 for the shown slice). The zero of energy was determined as the potential energy surface value at $r = 2.0\,a_0$ and $R = 1000\,a_0$.

merit. To this end, we average $\Delta(E_{\mathrm{shift}})$ over the interval $\left[\min(0, E_{\mathrm{shift}}^{(\mathrm{opt})}), \max(0, E_{\mathrm{shift}}^{(\mathrm{opt})})\right]$, i.e.,

$$\mathcal{F} = \frac{\int_0^{E_{\mathrm{shift}}^{(\mathrm{opt})}} dE_{\mathrm{shift}} \Delta(E_{\mathrm{shift}})}{\int_0^{E_{\mathrm{shift}}^{(\mathrm{opt})}} dE_{\mathrm{shift}}} \,. \tag{S3}$$

For a more fine-grained quantification of the differences, it is instructive to divide up kinetic energy histograms into windows $w = [w_{\min}, w_{\max}]$ corresponding to distinct peaks, with $\mathcal{W} = \{w\}$, the set of all windows. Unsigned integral deviations for matching theory and experiment on a per peak basis are given by [3]

$$\chi^{(w)} = \frac{\int_{w_{\min}}^{w_{\max}} dE_{\mathrm{kin}} \left(\sigma_{E_{\mathrm{shift}}}(E_{\mathrm{kin}}) - \sigma_{\mathrm{exp}}(E_{\mathrm{kin}})\right)}{\int_{w_{\min}}^{w_{\max}} dE_{\mathrm{kin}} \sigma_{\mathrm{exp}}(E_{\mathrm{kin}})} \,. \tag{S4}$$

The deviations in Eq. (S4) take into account the shapes of the individual peaks and not just their maximum intensities.

III. CROSS SECTION FOR INITIAL VIBRATIONAL EXCITATION $v = 2$

Figure S4 shows the kinetic energy spectra obtained with the three PESs, for initial wavepackets with $v = 2$, convoluted with the experimental resolution, and shifted along the kinetic energy axis to minimize the root-mean-square (RMS) difference between computed and observed peak positions. It complements the data for $v = 1$ shown in Fig. 1 in the main text. Note that for $v = 2$ differences between the three PES are sufficiently large to remain visible even after convolution, most noticeably so for the peak around $600\,\mathrm{cm}^{-1}$ in Fig. S4 corresponding to the $v = 2, j = 1 \to v' = 1, j' = 7$ transition.

Similarly, Fig. S5 shows the cross section as a function of the total energy for a double initial vibrational excitation, in analogy to Fig. 2 for $v = 1$. The $v = 2$ cross section shows the same variation in FR structure observed in $v = 1$ but in contrast to a single vibrational excitation, most FRs appear as broader, less pronounced peaks.

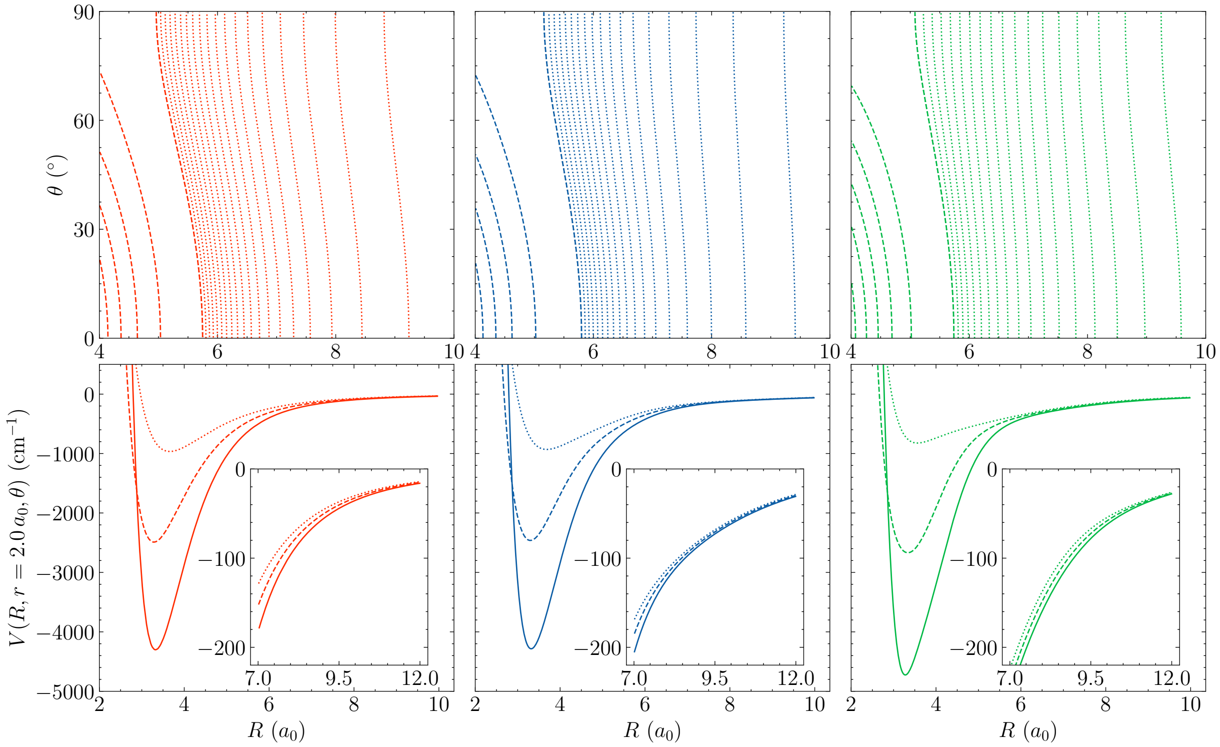

FIG. S2. Comparison of PESs focusing on the long-range part. Top panels from left to right: PESs CCSD(T)-5 , MRCI-5 and MRCI-4 for $R \in [4, 10]$ a$_0$ and $r = 2.0\,a_0$. With increasing R the anisotropy for MRCI-5 reduces considerably compared with the other two PESs. Spacing between dashed line contours is 500 cm^{-1} as before whilst the spacing between dotted contours is 25 cm^{-1}. Bottom panels: selected one dimensional cuts for $\theta = 0°$ (solid), 45° (dashed) and 90° (dotted lines), with $r = 2.0\,a_0$. Inset panels show a close up of the long range behaviour, emphasising the differences in both spread and anisotropy even at large R. As above, the CCSD(T)-5 , MRCI-5 and MRCI-4 contours and graphs are shown in red, blue and green respectively.

IV. OPTIMIZED SCALING

Experimental noise leads to a certain portion of the cross section appearing where it should not (i.e., in between peaks, as can be seen for kinetic energies in the range of 200 cm^{-1} and 500 cm^{-1}, for instance). As such, the scaling technique of Eq. (S1) leads to a slight overestimation of the theoretical peak heights relative to the experimental ones (because a higher proportion of the overall area of the cross section curve is concentrated on the peaks). By simultaneously optimising for both the scale and the shift in energy according to

$$\begin{aligned}\Delta_{\text{opt}} &= \min_{b,E_{\text{shift}}} \Delta(b, E_{\text{shift}}) \qquad \text{(S5)}\\ &= \min_{b,E_{\text{shift}}} \Bigg(\int_{\mathbb{R}_+} dE_{\text{kin}} \\ &\quad \times |\sigma_{\text{exp}}(E_{\text{kin}}) - b\tilde{\sigma}_{\text{theory}}(E_{\text{kin}} + E_{\text{shift}})|^2 \Bigg)^{-1/2},\end{aligned}$$

we can find the best possible scale for the simulated cross section in the RMS deviation sense. Above, we have $a = 1/\int dE_{\text{kin}}\tilde{\sigma}_{\text{exp}}(E_{\text{kin}})$ and the rescaled theoretical cross section is determined by the new, optimised parameter b.

The cross sections as a function of kinetic energy scaled with the optimised scaling parameter are shown in Figs. S6 and S7 for $v = 1$ and $v = 2$ respectively. At the cost of additional complexity, these reveal an overall better fit with the experimental cross section curve in terms of the various peak heights when compared to their 'normalised' counterparts. Conversely, the peaks in the simulated cross sections with 'normalised scaling' shown in Figs. 1 and S4 demonstrate a clear trend of overshadowing their measured counterparts, mostly notably for the $j' = 3, 5$ and 7 peaks in the case of the CCSD(T)-5 and MRCI-5 PES. Table S1 shows the difference that the chosen scaling scheme makes in terms of the figure of merit $\mathcal{F}$, (energy-shifted) RMS deviation $\min_{E_{\text{shift}}} \Delta(E_{\text{shift}})$ and optimal energy shift $E_{\text{shift}}^{(\text{opt})}$. Significantly, for $v = 2$, it is seen that in the optimised scaling scheme, the CCSD(T)-5 PES outperforms the MRCI-4 PES when it comes to $\min_{E_{\text{shift}}} \Delta(E_{\text{shift}})$. In contrast, when using the scaling of Eq. (S1), the energy shifted RMS deviation $\min_{E_{\text{shift}}} \Delta(E_{\text{shift}})$ for $v = 2$ was minimal for the MRCI-4 PES. In this case the most notable difference is in the overestimation of the height of the peak comprised of the $v' = 1$ and $j' = 2, 3$ as well as the $v' = 0$ and $j' = 9$ final states, as well as the peak due to $v' = 1$ and $j' = 7$ for the CCSD(T)-5 PES. It is seen that optimised rescaling causes the energy shifted RMS deviation

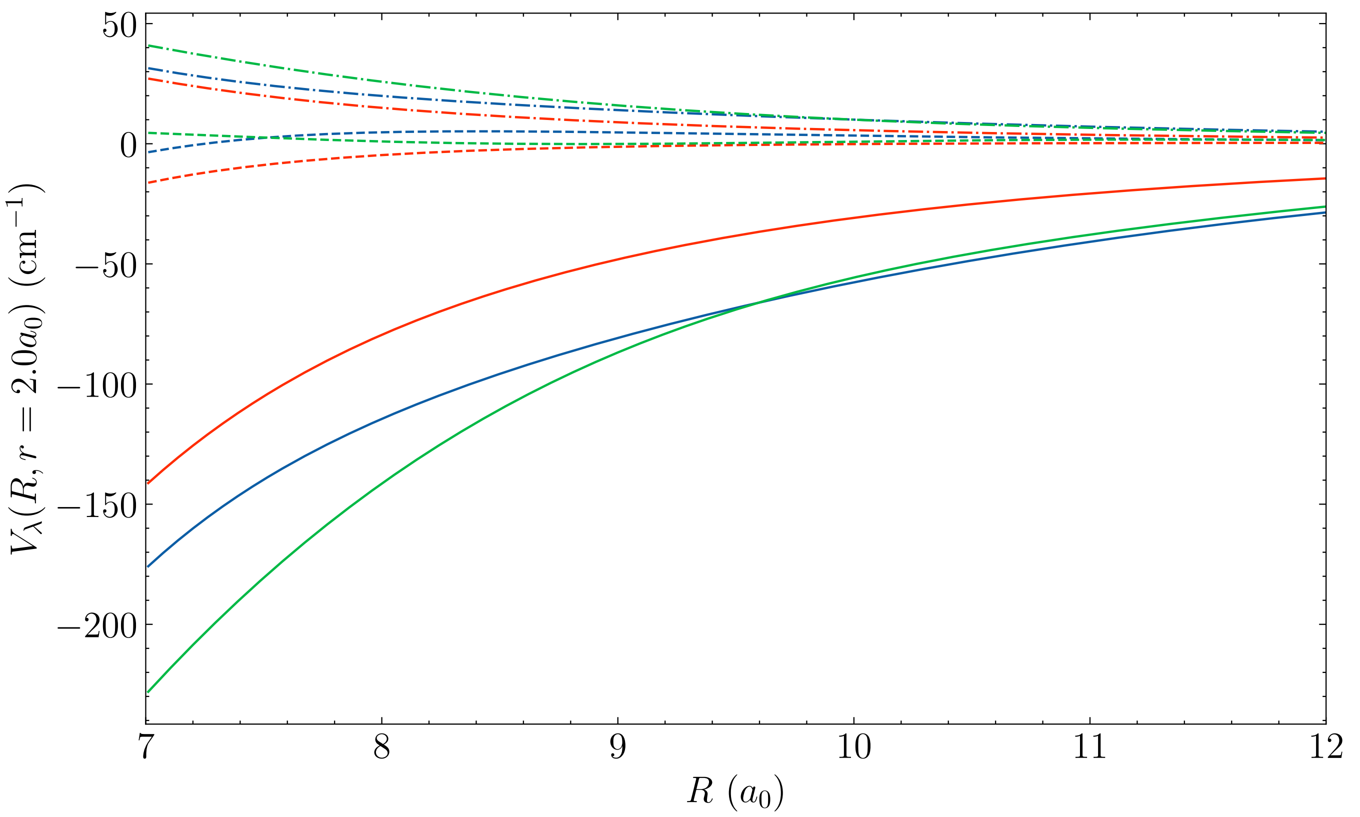

FIG. S3. Comparison of the long range behaviour of the various potential energy surfaces, portrayed in terms of the R-dependent Legendre coefficients $V_\lambda(R, r)$ for a fixed equilibrium distance $r = 2$ a$_0$. Large deviations are seen between the isotropic $\lambda = 0$ curve for CCSD(T)-5 and those corresponding to the other two potential energy surfaces. The solid, dashed and dash-dotted lines correspond to $\lambda = 0, 2$ and 4 respectively.

to drop by $1.8 \cdot 10^{-3}$ in the case of CCSD(T)-5 , whilst the improvement for the MRCI-4 PES is more moderate at $0.3 \cdot 10^{-3}$. The figure of merit $\mathcal{F}$ is affected in a similar way, with a larger margin of improvement observed for CCSD(T)-5 , causing it to more clearly outperform the other PES when utilising optimised scaling.

V. REGULARISATION

We have accounted for the presence of noise in the experimental cross section by investigating whether a regularisation technique, similar to that employed in [7], influences the results. Regularisation was implemented by subtracting δ_{reg} from the difference in cross sections, whilst ensuring the kinetic energy-dependent difference never drops below zero

$$\epsilon(E_{\text{kin}}) = \max\left(|\sigma_{\text{exp}}(E_{\text{kin}}) - \sigma_{\text{shift}}(E_{\text{kin}})| - \delta_{\text{reg}}, 0\right) , \tag{S6}$$

where $\sigma_{\text{shift}}(E_{\text{kin}})$ is the shifted and rescaled simulated cross section under either scaling method. The non-regularised difference between cross sections in the expressions for $\Delta(E_{\text{shift}})$ can then be replaced with the regularised expression $\Delta_{\text{reg}}(E_{\text{shift}})$. Whilst it was observed that regularisation does indeed affect the RMS deviation and figure of merit (whose regularised counterpart is denoted by $\mathcal{F}_{\text{reg}}$) as summarised in Table S1, the cross sections due to all PES are affected in almost the same way, i.e., regularisation does not qualitatively impact the PES comparison results.

VI. DETAILS ON CONVOLUTION RESOLUTION SCALING

The decomposition of the unconvoluted cross section according to the total angular momentum J and initial partial waves ℓ (multiplied with -1 and offset for better visibility) is also shown in Fig. S8: Contributions with larger J and ℓ tend to occur at higher kinetic energies, most noticeably for para-H_2^+. As such, features present in the gold curves, corresponding to ten times the resolution, can be attributed to a particular J and ℓ contribution in many cases.

There are also initial channels whose contributions cannot be resolved, for example at 650 cm^{-1} for the CCSD(T)-5 PES in Fig. S8(g), which corresponds to a total energy of -20 cm^{-1} in Fig. 2(d).

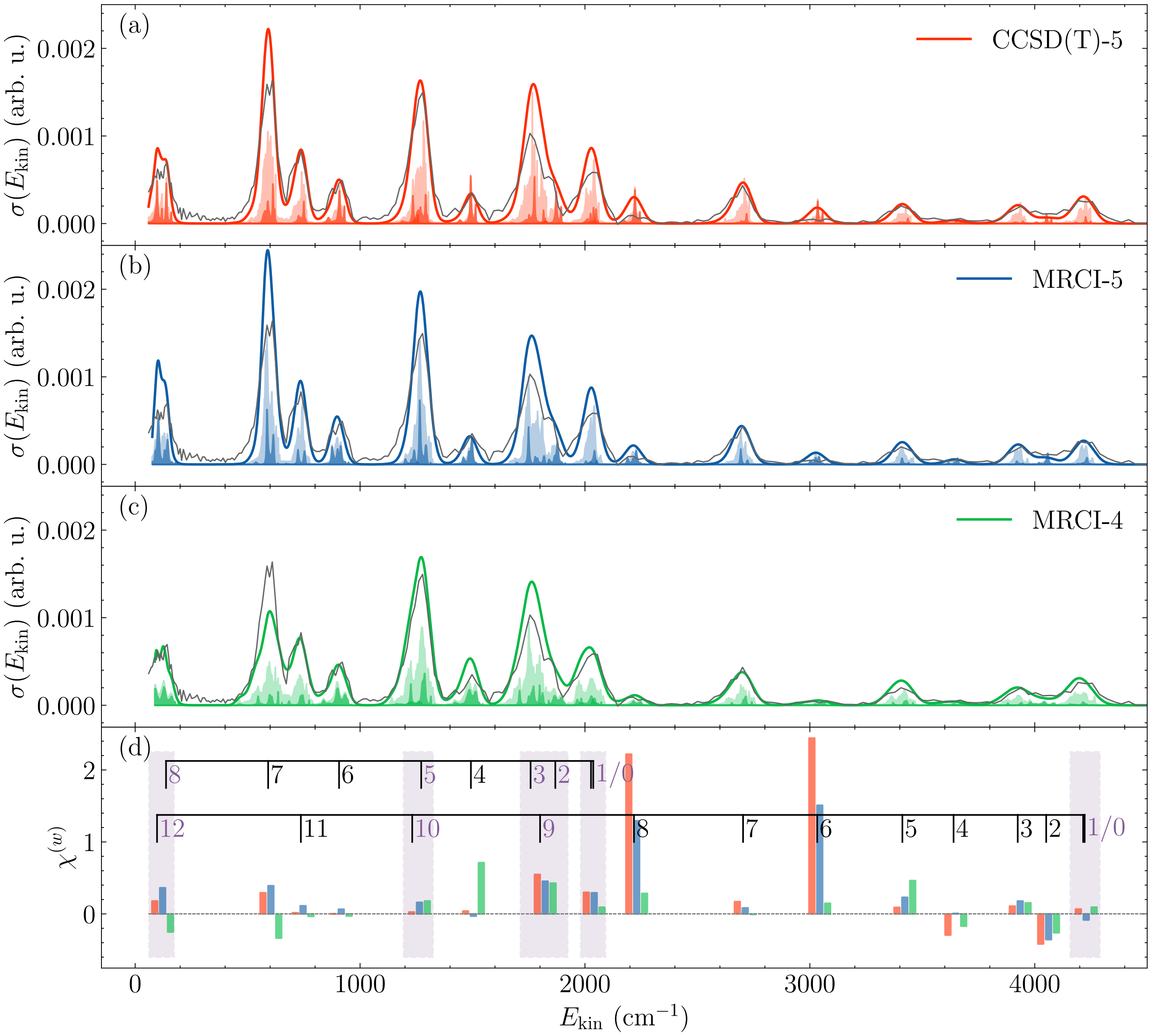

FIG. S4. Same as Fig. 1 but for the initial wavepacket in the diatomic rotational ground state and with $v = 2$. The unconvoluted cross sections have been scaled by a factor of three tenths relative to their convoluted counterparts.

[1] O. T. Unke and M. Meuwly, Toolkit for the construction of reproducing kernel-based representations of data: Application to multidimensional potential energy surfaces, Journal of Chemical Information and Modeling **57**, 1923 (2017).

[2] H.-J. Werner, P. J. Knowles, F. R. Manby, J. A. Black, K. Doll, A. Heßelmann, D. Kats, A. Köhn, T. Korona, D. A. Kreplin, *et al.*, The Molpro quantum chemistry package, The Journal of Chemical Physics **152**, 144107 (2020).

[3] B. Margulis, K. P. Horn, D. M. Reich, M. Upadhyay, N. Kahn, A. Christianen, A. van der Avoird, G. C. Groenenboom, M. Meuwly, C. P. Koch, and E. Narevicius, Tomography of Feshbach resonance states, Science **380**, 77 (2023).

[4] S.-J. Lv, P.-Y. Zhang, K.-L. Han, and G.-Z. He, Exact quantum scattering study of the Ne+H_2^+ reaction on a new *ab initio* potential energy surface, The Journal of Chemical Physics **132**, 014303 (2010).

[5] J. Xiao, C.-L. Yang, X.-F. Tong, M.-S. Wang, and X.-G. Ma, Quasi-classical trajectory study of the Ne+H_2^+ $\rightarrow$ NeH^++H reaction based on global potential energy surface, The Journal of Physical Chemistry A **115**, 1486 (2011).

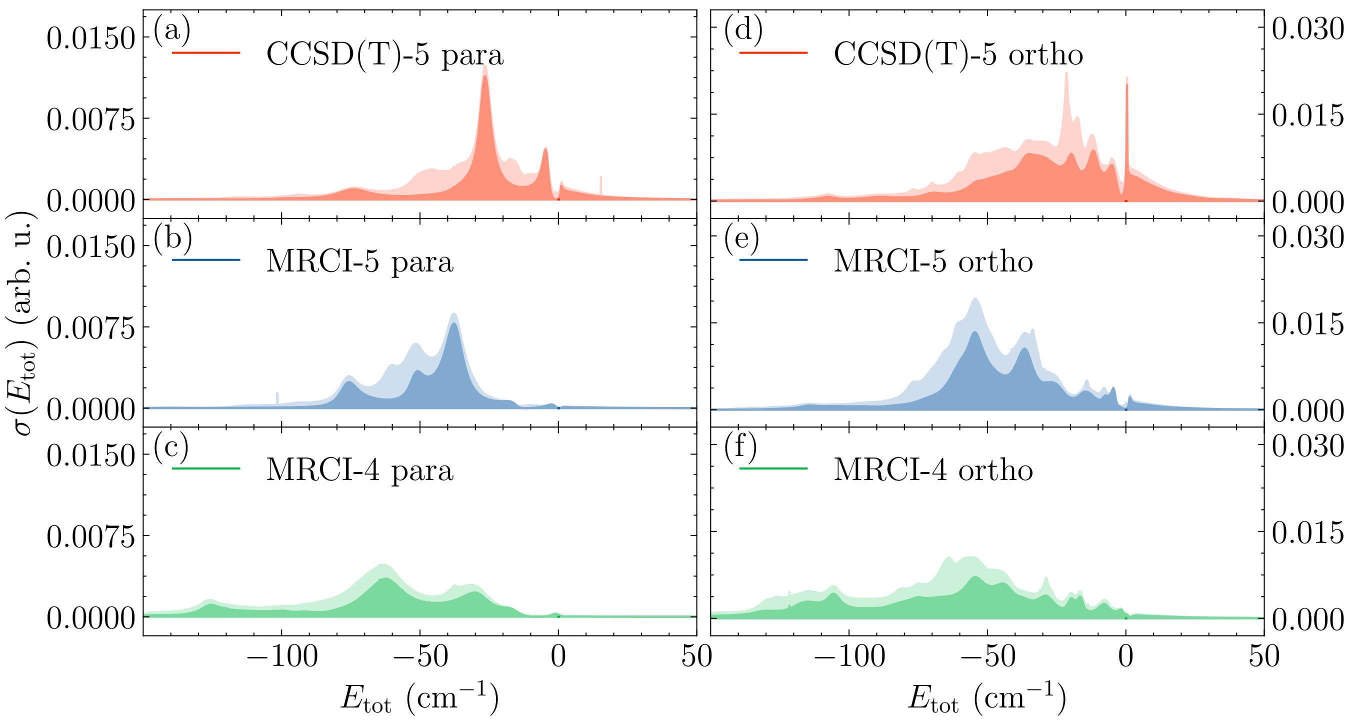

FIG. S5. Same as Fig. 2, but for $v = 2$.

v	scaling	PES	$\mathcal{F}$	$\mathcal{F}_{\mathrm{reg}}$	$\min_{E_{\mathrm{shift}}} \Delta(E_{\mathrm{shift}})$	$\min_{E_{\mathrm{shift}}} \Delta_{\mathrm{reg}}(E_{\mathrm{shift}})$	$E_{\mathrm{shift}}^{(\mathrm{opt})}$
1	Eq. (1)	CCSD(T)-5	$9.9 \cdot 10^{-3}$	$8.0 \cdot 10^{-3}$	$9.9 \cdot 10^{-3}$	$8.0 \cdot 10^{-3}$	$-0.74\mathrm{cm}^{-1}$
		MRCI-5	$1.2 \cdot 10^{-2}$	$1.0 \cdot 10^{-2}$	$1.0 \cdot 10^{-2}$	$8.1 \cdot 10^{-3}$	$21.0\mathrm{cm}^{-1}$
		MRCI-4	$1.1 \cdot 10^{-2}$	$9.4 \cdot 10^{-3}$	$7.9 \cdot 10^{-3}$	$6.0 \cdot 10^{-3}$	$30.0\mathrm{cm}^{-1}$
	Eq. (S1)	CCSD(T)-5	$7.4 \cdot 10^{-3}$	$5.1 \cdot 10^{-3}$	$7.4 \cdot 10^{-3}$	$5.1 \cdot 10^{-3}$	$-0.86\mathrm{cm}^{-1}$
		MRCI-5	$1.0 \cdot 10^{-2}$	$8.0 \cdot 10^{-3}$	$8.1 \cdot 10^{-3}$	$5.8 \cdot 10^{-3}$	$21.0\mathrm{cm}^{-1}$
		MRCI-4	$9.9 \cdot 10^{-3}$	$8.0 \cdot 10^{-3}$	$6.6 \cdot 10^{-3}$	$4.5 \cdot 10^{-3}$	$29.0\mathrm{cm}^{-1}$
2	Eq. (1)	CCSD(T)-5	$8.5 \cdot 10^{-3}$	$7.0 \cdot 10^{-3}$	$8.5 \cdot 10^{-3}$	$7.0 \cdot 10^{-3}$	$-0.54\mathrm{cm}^{-1}$
		MRCI-5	$1.1 \cdot 10^{-2}$	$9.1 \cdot 10^{-3}$	$9.7 \cdot 10^{-3}$	$8.3 \cdot 10^{-3}$	$16.0\mathrm{cm}^{-1}$
		MRCI-4	$9.0 \cdot 10^{-3}$	$7.6 \cdot 10^{-3}$	$7.3 \cdot 10^{-3}$	$6.1 \cdot 10^{-3}$	$27.0\mathrm{cm}^{-1}$
	Eq. (S1)	CCSD(T)-5	$6.7 \cdot 10^{-3}$	$5.0 \cdot 10^{-3}$	$6.7 \cdot 10^{-3}$	$5.0 \cdot 10^{-3}$	$-0.68\mathrm{cm}^{-1}$
		MRCI-5	$7.8 \cdot 10^{-3}$	$6.1 \cdot 10^{-3}$	$6.9 \cdot 10^{-3}$	$5.1 \cdot 10^{-3}$	$16.0\mathrm{cm}^{-1}$
		MRCI-4	$8.6 \cdot 10^{-3}$	$7.2 \cdot 10^{-3}$	$7.0 \cdot 10^{-3}$	$5.7 \cdot 10^{-3}$	$27.0\mathrm{cm}^{-1}$

TABLE S1. Expanded version of Table 1, including regularisation and the alternative cross section scaling scheme introduced in Eq. (S5). For both "initial" vibrational states, the PES that performs best for a given quantifier is highlighted, using the color code of Figs. 1 and S4.

[6] S. G. Johnson, The nlopt nonlinear-optimization package, http://github.com/stevengj/nlopt.

[7] K. P. Horn, L. I. Vazquez-Salazar, C. P. Koch, and M. Meuwly, Improving potential energy surfaces using experimental Feshbach resonance tomography, Science Adv. **10**, eadi6462 (2024), 2309.16491.

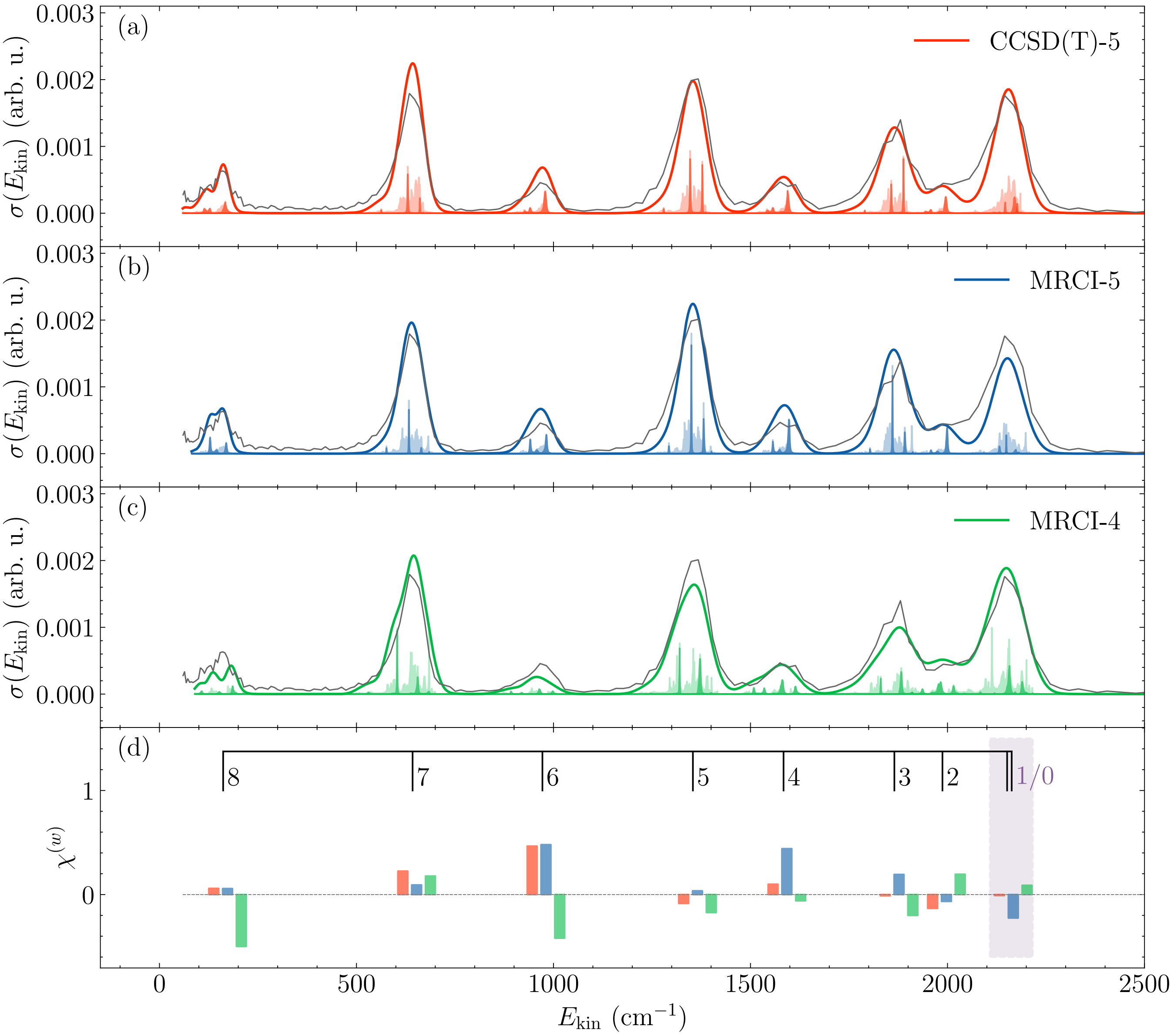

FIG. S6. Same as Fig. 1, but with the fitted relative scaling of Eq. (S5).

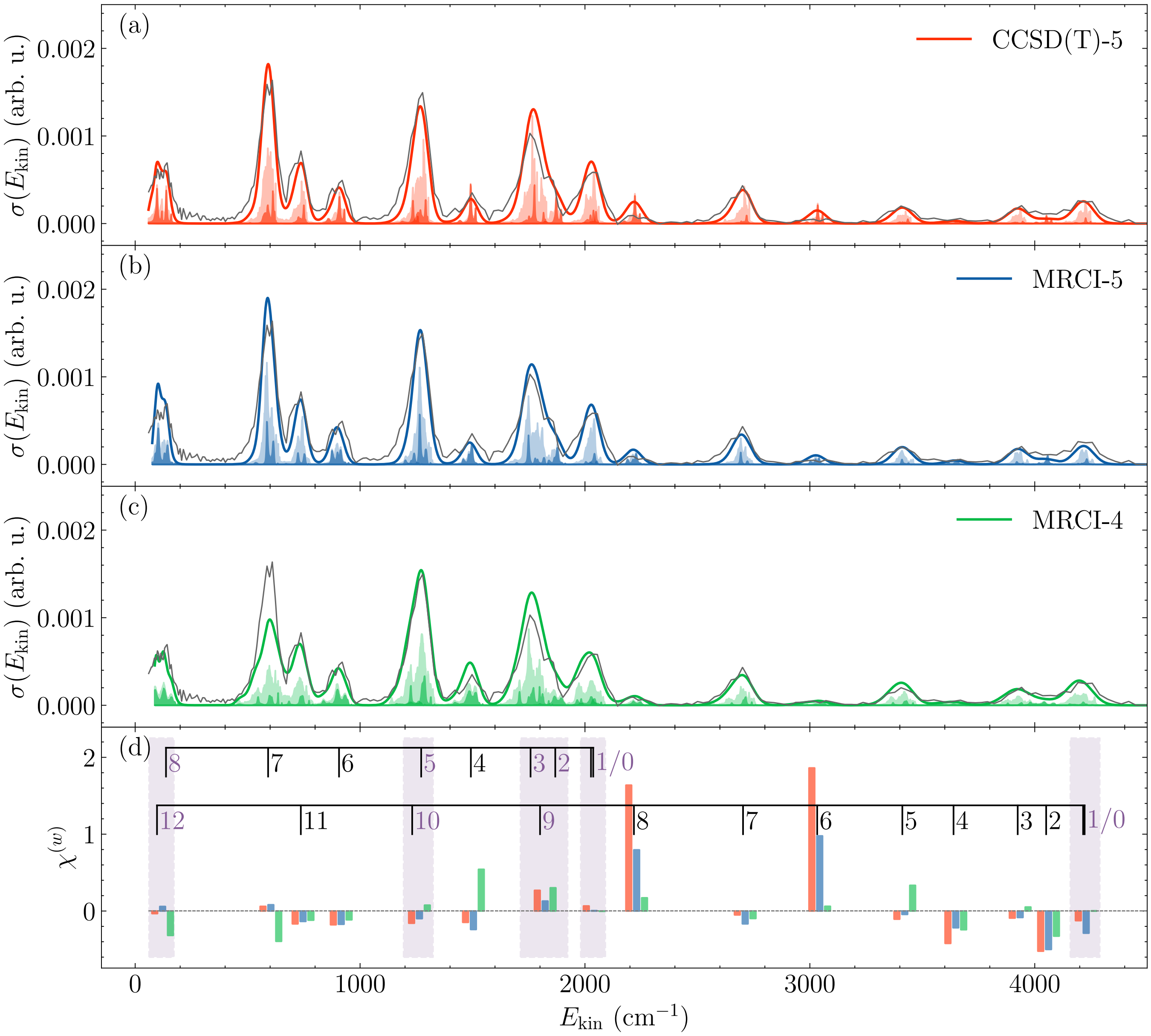

FIG. S7. Same as Fig. S4, but with the fitted relative scaling of Eq. (S5).

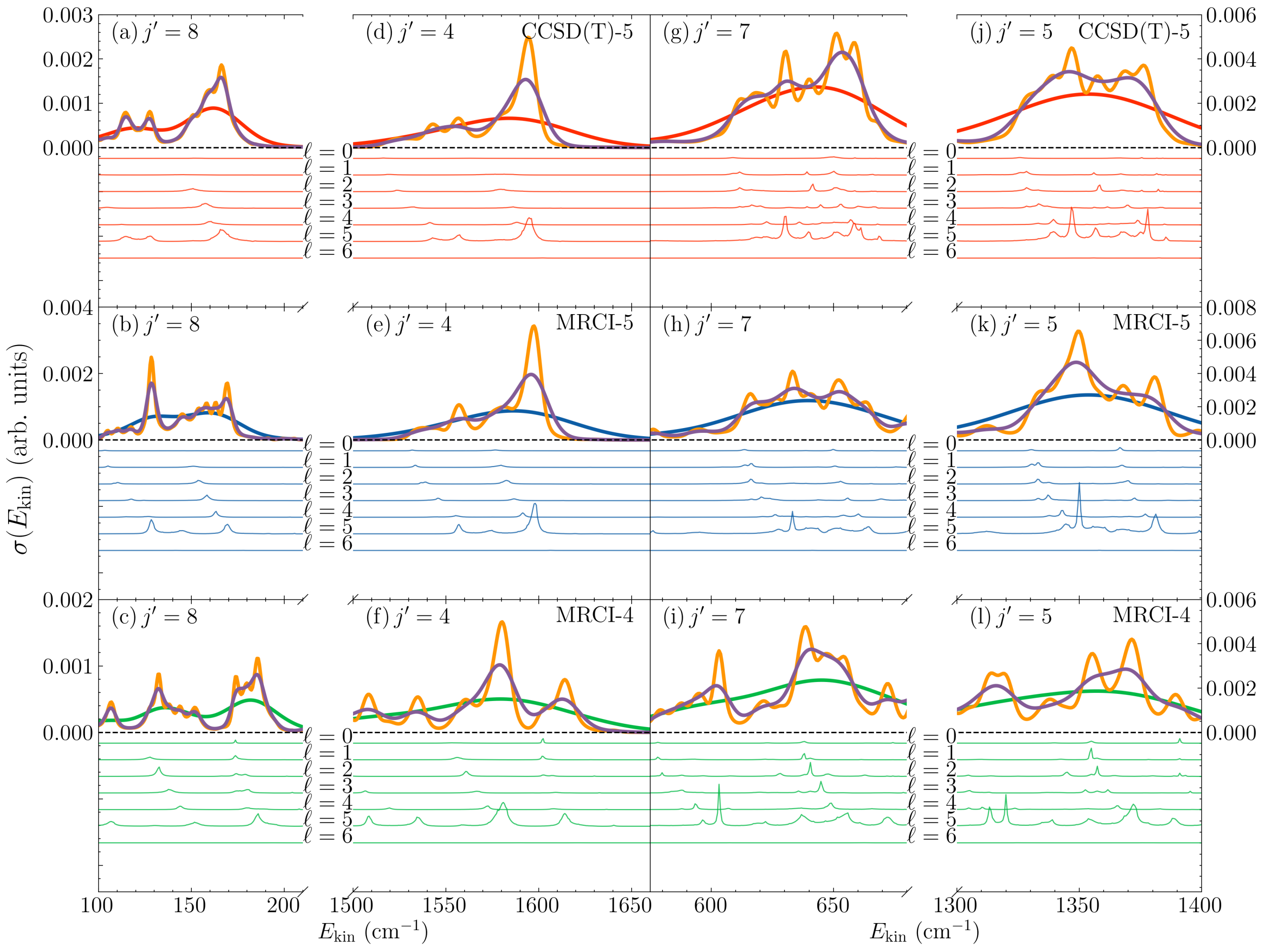

FIG. S8. Role of convolution width in the calculated cross sections for $v = 1$, using a convolution of fixed width for all energies (thick solid lines): Peaks appearing broad in Fig. 1 at the original convolution width start to show substructure when improving the (fixed-width) energy resolution by a factor of four (purple), resp. 10 (gold). Also shown are the individual contributions of the various initial channels (thin solid lines).
